# Smartphone-Assisted Phenol–Sulfuric Acid Assay with ImageJ Analysis for Total Carbohydrate Quantification

**DOI:** 10.3390/molecules31213794

**Published:** 2026-10-22

**Authors:** Zaiba Iqbal, Gordon A. Morris

**Affiliations:** Department of Physical and Life Sciences, School of Science, Computing and Engineering, University of Huddersfield, Huddersfield HD1 3DH, UK; zaiba.iqbal@hud.ac.uk

**Keywords:** phenol–sulfuric acid (PSA) assay, total carbohydrate content, smartphone-assisted PSA assay, ImageJ, pectin

## Abstract

The phenol–sulfuric acid (PSA) assay is widely recognized for its versatility and simplicity in the detection and quantification of carbohydrates. However, as well as the appropriate reagents, this method requires a UV–visible spectrophotometer, the cost of which may be prohibitive for some research groups. This study aims to revisit the PSA assay and investigate a novel smartphone-assisted method as a potential alternative to using a spectrophotometer. To the best of our knowledge this is the first time the PSA assay has been used in this way. A range of monosaccharides—glucose, fructose, galactose, xylose, arabinose, mannose, fucose, rhamnose, and galacturonic acid—were tested to establish calibration curves and evaluate the PSA assay’s quantitative parameters, utilizing both the traditional spectrophotometer-based method and the adapted smartphone-assisted assay, where ImageJ was used to estimate the change in “colour” of the samples. Calibration curves generated from the traditional method demonstrated excellent linearity, with adjusted coefficients of determination (Adj. R^2^) between 0.992 and 0.998; the slopes (m), intercepts (c) and the standard deviation of the residuals of these curves (s_x/y_) were used to estimate the limits of detection (LOD) and limits of quantification (LOQ) at 95% confidence. For the traditional PSA assay, the LOD values ranged from 5.5 to 12.3 ppm and the LOQ values from 16.8 to 37.5 ppm. Differences in the LOD/LOQ between different monosaccharides were attributed to variations in chemical reactivity, molecular structure, and chromophore absorbance at 490 nm. The smartphone-assisted PSA assay with ImageJ also demonstrated excellent linearity, with adjusted coefficients of determination (Adj. R^2^) between 0.991 and 0.997. The LOD values ranged from 7.9 to 13.1 ppm and the LOQ values from 24.1 to 39.9 ppm, providing an alternative for use in resource-limited environments. To demonstrate the potential usefulness of this assay, two case studies were undertaken: (i) to estimate the total carbohydrate content of pectin-like polymers from okra and potato, using both the traditional and smartphone-assisted PSA assay with ImageJ; and (ii) to generate a chromatogram in the preparative size exclusion chromatography (SEC) of a commercial citrus pectin sample. In the first case study, the total carbohydrate contents across all samples were similar using both methods and in the second case study this allowed the analysis of 32 samples (in triplicate) in one image. Future work should focus on fully optimising both image capture and the analysis steps of the new protocol and extending the method’s applicability to complex biological and environmental matrices. Overall, this study offers an alternative to the traditional PSA assay, reinforcing its role as a fundamental tool in modern carbohydrate quantification.

## 1. Introduction

Carbohydrates, a major class of biomolecules, are ubiquitous in living organisms and play critical roles in structural integrity, energy metabolism, and cell signalling [1]. Their presence in natural and processed products underlines the necessity of accurate and efficient quantification methods [2,3] in sectors such as biomedicine, pharmaceuticals, nutrition, food technology, and environmental research [4,5].

Over the years, the complexity and diversity of carbohydrate-containing matrices have led to the development of numerous analytical techniques. High-performance liquid chromatography (HPLC), gas chromatography (GC), nuclear magnetic resonance (NMR) spectroscopy, capillary electrophoresis (CE), and infrared spectroscopy (IR) are among the sophisticated methods routinely employed for carbohydrate analysis [6,7,8,9]. These techniques offer high sensitivity and selectivity, especially when resolving complex mixtures of mono-, oligo-, and polysaccharides. Nevertheless, their utility is often limited by high operational costs, extensive sample preparation, long analysis times, and the requirements for skilled personnel and specialized infrastructure [10]. Consequently, these constraints can impede routine carbohydrate monitoring, particularly in settings with limited resources or where high-throughput analyses are necessary [11].

There is a continuing need for simpler, faster, and more cost-effective carbohydrate quantification tools. Examples of these types of analysis include colorimetric methods, e.g., the phenol–sulfuric acid (PSA) assay. These assays are based on the interaction of carbohydrates/monosaccharides with a chromogenic reagent, for example, phenol, under acidic conditions. This leads to the formation of coloured products that can be quantified by measuring their absorbance in the visible or near-UV region [12].

The PSA method, first developed by DuBois et al. (1956), has become the most cited and widely applied colorimetric approach for total carbohydrate determination [13]. According to the Web of Science^®^, over 35,000 publications reference the PSA method, testament to its enduring relevance and widespread acceptance in modern analytical chemistry [14].

**Figure 1:**
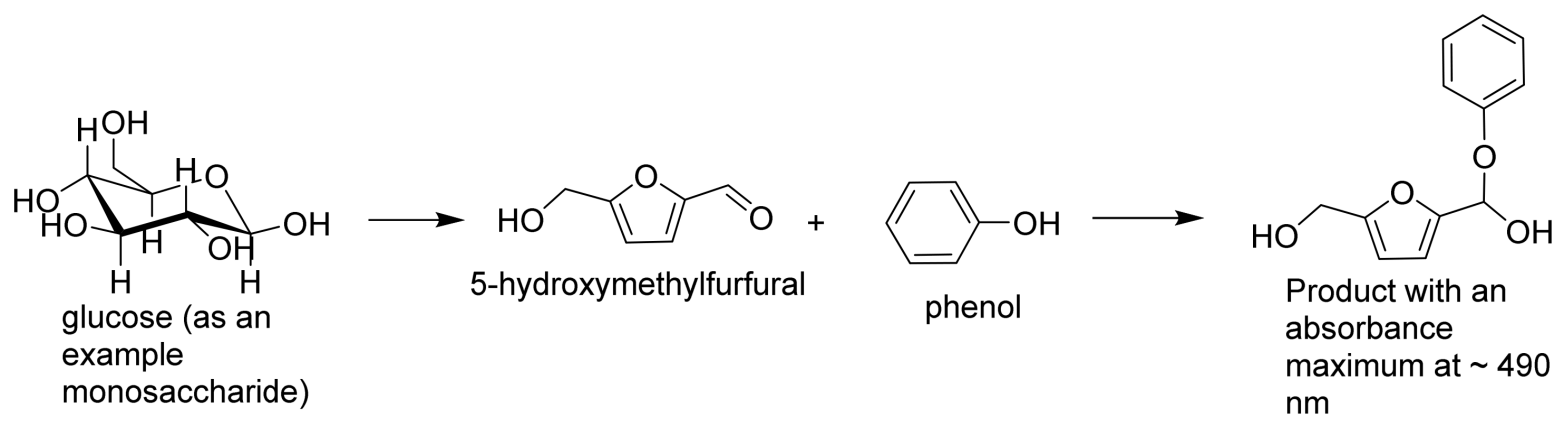
A reaction scheme showing an example reaction of phenol–sulfuric acid assay with glucose (adapted from [16]).

The fundamental chemistry underpinning this method involves the acid-catalysed dehydration of carbohydrates to form furfural (pentoses) or hydroxy-methyl furfural (hexoses) intermediates, which subsequently react with phenol to yield a chromogenic complex (Figure 1). The absorbance of this coloured product, typically measured at ~490 nm, is directly proportional to the total carbohydrate concentration [15]. The technique is notable for its simplicity, adaptability to microplate formats, compatibility with a wide range of carbohydrate structures (mono-, oligo-, and polysaccharides), and relatively low equipment cost requirements [12].

Central to any analytical method’s reliability are its limit of detection (LOD) and its limit of quantification (LOQ). In various industrial and research applications, such as quality control of low-sugar food products, detection of trace-level saccharides in fermentation media, and polysaccharide degradation, studies’ methods must reliably detect and quantify carbohydrates at minimal concentrations [12]. Historical data show that in automated systems using the phenol–sulfuric acid assay, the limits of detection for sugars in physiological fluids are lower than 1 ppm (1 µg/mL) [2], although there is a large variation in the published values of both LOD (0.05–15.1 ppm) and LOQ (0.14–49.8 ppm) [17,18,19,20,21,22,23,24,25,26]. However, the analytical performance of the phenol–sulfuric acid (PSA) assay is influenced by the experimental conditions under which the method is applied, including reagent concentrations, reaction time and temperature, detection wavelength, sample matrix, and monosaccharide composition [3,13].

This study seeks to address the need for a greater understanding of the phenol–sulfuric acid assay by

1. Determining accurate LOD and LOQ values for the PSA assay.
2. Comparing the traditional PSA method with a smartphone-assisted phenol–sulfuric acid assay with ImageJ analysis (NIH, Bethesda, MD, USA).

As 490 nm corresponds to the visible (orange) part of the electromagnetic spectrum, there is potential for these samples to be analysed using a standard digital camera or smartphone camera, with the use of ImageJ software (NIH, Bethesda, MD, USA) to estimate the “colour” or change in colour rather than a UV-vis spectrophotometer. It is expected that this novel adaptation of a classical carbohydrate chemistry may be particularly useful for field studies or where financial restrictions limit the use of analytical instrumentation. Other advantages include the potential to analyse a large number of samples in a single image. The expected outcomes of this investigation include a cheaper, easier PSA method developed using a mobile device with a camera and ImageJ software (NIH, Bethesda, MD, USA).

## 2. Materials and Methods

### 2.1. Materials

The study utilized an array of carbohydrate standards representative of various structural and functional classes, including pentoses (L-arabinose and D-xylose), hexoses (D-galactose, D-glucose, D-fructose and D-mannose), deoxyhexoses (L-fucose and L-rhamnose) and uronic acids (D-galacturonic acid). These carbohydrates were selected based on their prevalence in biological systems and food products. Phenol (C_6_H_6_O), concentrated sulfuric acid (H_2_SO_4_), and the commercial pectin sample were obtained from Sigma-Aldrich (Gillingham, UK).

### 2.2. Instrumentation

Spectrophotometric analysis was conducted using a Bio Wave II UV–Visible spectrophotometer (Biochrome, Cambridge, UK). UV transparent plastic cuvettes with a 1 cm path length were used [27]. Additionally, a vortex shaker (SciQuip Vortex VariMix, Rotherham, UK) was employed at each stage of sample preparation and reagent mixing to thoroughly mix solutions. This ensured uniform distribution of reactants and reduced the risk of concentration gradients or incomplete reactions that could compromise the reproducibility of the assay, which is common in the phenol–sulfuric acid assay due to the density of the sulfuric acid (~1.84 g cm^−3^ at room temperature).

### 2.3. Sample Preparation

#### 2.3.1. Phenol Stock Solution

Using an analytical balance, a 5% (*w*/*v*) phenol stock solution was freshly prepared. The solution was thoroughly mixed using a vortex mixer to ensure complete solubilization and homogeneity and stored at room temperature for use in subsequent assays.

#### 2.3.2. Carbohydrate Stock Solutions

Using an analytical balance with precision to four decimal places, 0.1 g of each carbohydrate standard (glucose, fructose, mannose, etc.) was weighed and dissolved in deionized water in a 100 mL volumetric flask. The solution was brought to volume and mixed thoroughly using a vortex shaker to achieve homogeneity. Each stock solution had a final concentration of 1000 ppm.

#### 2.3.3. Traditional Phenol–Sulfuric Assay

From the prepared 1000 ppm stock solutions, standard working solutions were prepared to obtain standards of six concentrations, 0, 20, 40, 60, 80, and 100 ppm. The colorimetric assay was performed by adding exactly 0.2 mL of each standard into separate test tubes. A quantity of 0.2 mL of freshly prepared 5% (*w*/*v*) phenol solution was then added to each test tube containing standard solutions. Subsequently, 1 mL of concentrated sulfuric acid was introduced rapidly and directly into each mixture, and the resultant solutions were immediately mixed using a vortex shaker to ensure homogeneity and minimize local overheating, which could lead to inconsistent colour development or sample degradation. The reaction mixtures were allowed to stand at ambient temperature (~25 °C) for a fixed period of 10 min, after which the colour had fully developed [3]. Spectrophotometric measurements were conducted immediately following the reaction period. Each reaction mixture was then transferred into a clean plastic cuvette, and absorbance was recorded at 490 nm. This wavelength was selected based on the absorption maximum for the phenol–furfural chromophore complex, as established in earlier studies [13].

All absorbance readings were carried out in triplicate as independent measurements at all concentrations for each monosaccharide, to ensure statistical reliability. Variability between replicates was assessed using standard deviation.

#### 2.3.4. Calibration Curves

The absorbance values obtained for each monosaccharide concentration were plotted against their known concentrations in order to construct calibration curves. Linear regression analysis was performed to derive the equation of the line in the form y = mx + c, where “y” denotes absorbance, “m” is the slope, “x” is the concentration, and “c” is the y-intercept. The correlation coefficient (Adj. R^2^) was calculated to evaluate the linearity of the response.

##### Determining the Limit of Detection (LOD) and Limit of Quantification (LOQ)

Two important parameters that can be estimated from a calibration curve are the limit of detection (LOD) and the limit of quantification (LOQ). They are estimated using the slope (m), intercept (c), the standard deviation of the residuals (s_y/x_) and the uncertainty threshold (k), which relates to the required confidence limits [28]. In this study, they were estimated using Equations (1) and (2) for the calculation of LOD and LOQ, respectively:

𝑦_𝐿_ = c + 𝑘𝑠_y/x_ (1)

𝑥_𝐿_ = (𝑦_𝐿_ − 𝑐)/𝑚 (2)

where y_L_ is the minimum detectable signal, c is the intercept of the line of best fit, *k* is the uncertainty threshold (3.28 * and 10, respectively (95% confidence), for LOD and LOQ), *s*_y/x_ is the standard deviation of the residuals [29] and *m* is the slope. The terms s_y/x_, *c* and *m* can be found using the LINEST function in MS Excel. Where *x_L_* is the limit of detection, when *k* = 3.28 * in Equation (1); and it is the limit of quantification, when *k* = 10 in Equation (1). * As this is 2 × 1.645, it can be rounded to 3.28, 3.29 or 3.3.

### 2.4. Image J Software for Colour Detection

**Figure 2:**
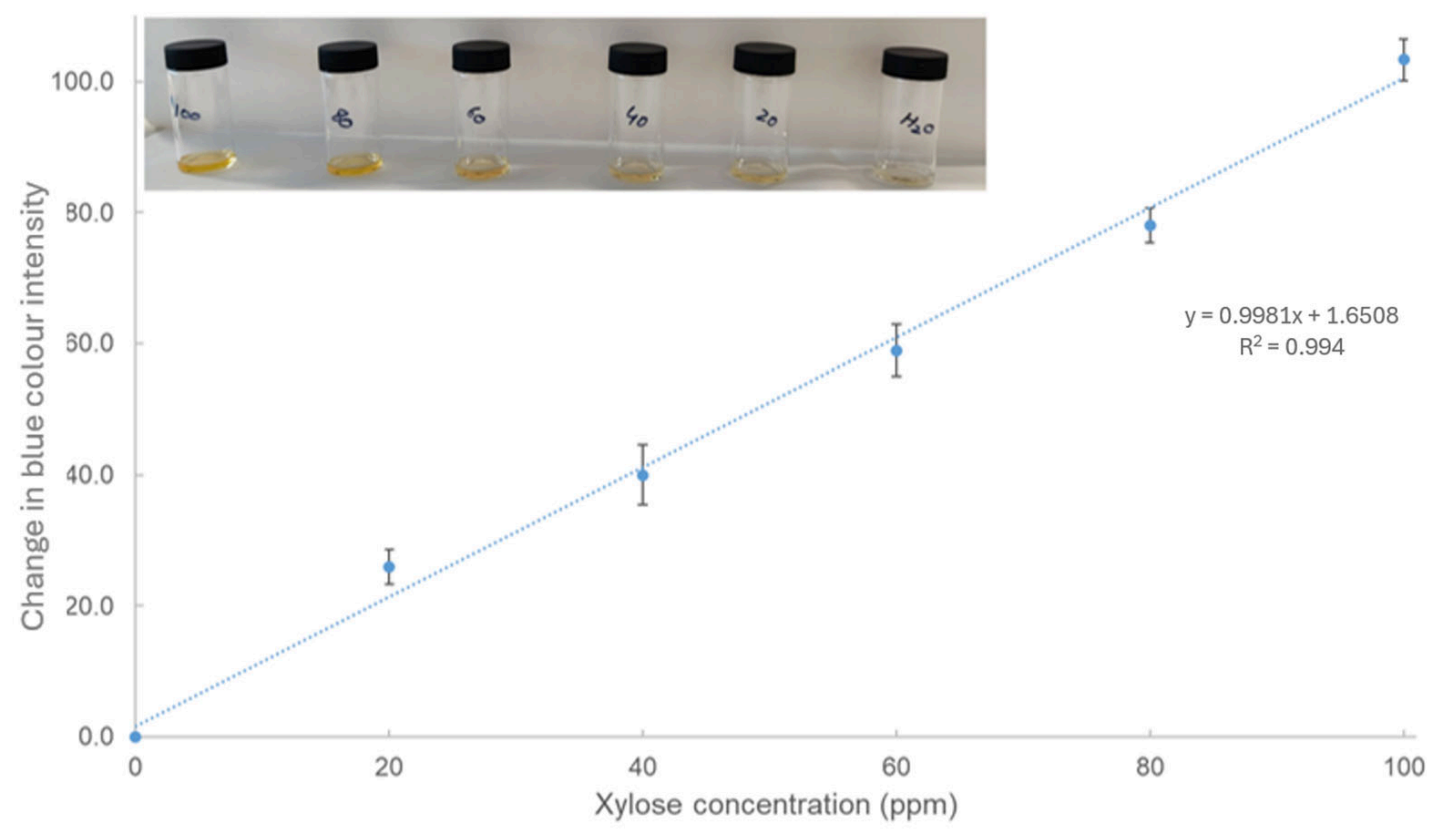
The change in “blue” colour for xylose upon reaction with the phenol–sulfuric acid assay. Inset: colour responses of various concentrations for xylose (increasing in concentration from right to left, the numbers visible on the vials are the concentrations in ppm, water is a blank (0 ppm)) in the solution system based on phenol–sulfuric acid assay. The values are the mean of independent measurements in triplicate, and the error bars represent ±1 standard deviation of the mean.

**Figure 3:**
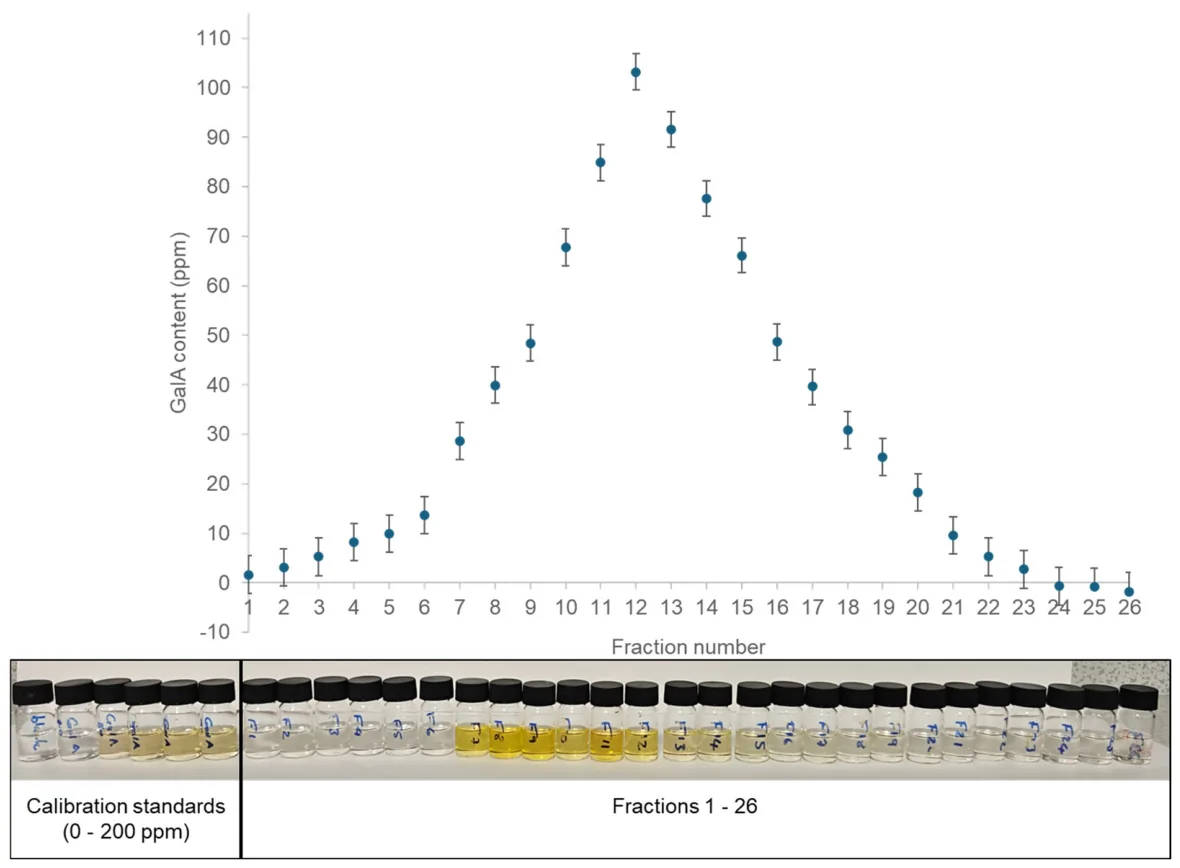
The size exclusion chromatogram of the commercial citrus pectin sample, used as an example polysaccharide. The pectin was separated into 26 fractions (5 mL) and an aliquot of this was used in the new smartphone-assisted phenol–sulfuric acid assay with ImageJ analysis. The raw data is shown below the chromatogram—the calibration standards are on the left and vial containing each fraction is below its corresponding galacturonic acid concentration in the chromatogram. Errors represent ±1 standard deviation (n = 3).

Samples were prepared as in Section 2.3.3. After the colour had fully developed, high-resolution images of the sample solutions at different concentrations (0–100 ppm) were captured, in single frames, using a smartphone OnePlus Model (9R) processor (Qualcomm^®^ Snapdragon TM 870 Octa-core) under controlled lighting conditions to ensure uniform illumination. The smartphone was held by hand, approximately 1.0 m from the sample vials. The vials were arranged in a row on the same matte white paper sheet under white laboratory LED lighting at room temperature, and another matte sheet of paper was placed behind the vials (see insets in Figure 2 and Figure 3). The camera settings were ISO 400, exposure time 1/100 s, aperture f/1.8 and focal length 24 mm (full-frame equivalent), with autofocus. Flash, HDR/AI scene enhancement and zoom were switched off. Images were saved as JPEG files at a resolution of 4096 × 3072 pixels (approximately 4.7 MB) and cropped before analysis to remove excess background, without altering the image dimensions. Each digital image was analysed using ImageJ software (Version 1.54g, National Institutes of Health, Bethesda, MD, USA; https://imagej.net/ij/) and converted to RGB (red, green, and blue) format before the individual colour channels were separated for analysis. The RGB intensity values were extracted and preliminary analysis of the data demonstrated that the blue channel was of greatest interest. As blue is the opposite colour on the colour wheel to orange, a decrease in blue colour should reflect an increase in orange colour. Blue colour (mean intensity) values on an 8-bit scale (0–255) for each monosaccharide standard were determined by selecting the “Measure” function on the ImageJ toolbar and drawing a fixed rectangular region of interest (ROI) measuring 807 × 199 pixels on each image [30]. The same ROI dimensions were used for all measurements, with the ROI positioned over the liquid region of each vial. The changes in intensity (Equation (3)) of the “blue colour” were plotted against concentration to generate calibration curves, the line of best fit was plotted and the LOD and LOQ were estimated as described in the section entitled Determining the Limit of Detection (LOD) and Limit of Quantification (LOQ), where in this case, “y” denotes “change in blue colour”, and “x” is the concentration. This is similar to methods previously used for the determination of lead ions [30], melamine [31], blue food dye, sports drinks, and iron (III) chloride [32].

Δ (blue colour) = mean intensity (blank) − mean intensity (sample)(3)

For example, if the mean blue intensity of the glucose blank = 190.4 and the mean blue intensity of the 40 ppm standard was 162.4, then the change (D) in intensity was 28.0.

### 2.5. Practical Applications of the New Smartphone Image-Based Assay

In order to determine the usefulness of this novel smartphone-based method, two case studies were conducted: (i) comparisons of this novel method with the standard phenol–sulfuric acid for polysaccharide extracts (Section 2.5.1) and (ii) as part of a preparative size exclusion separation of a commercial pectin sample (Section 2.5.2).

#### 2.5.1. Total Carbohydrate Contents of Polysaccharides

Okra polysaccharides [1,2,3,4,5,7,9,10,11] were extracted from okra pods, as described previously [33,34,35], at pH 6.0 and 80 °C. Okra polysaccharide (OP) 6 and potato pectin (PP) were was extracted with hot water 80 °C [36,37] and OP8 was extracted with cold water at room temperature, ~25 °C. Quantities of 0.2 mL of the okra (OP) and potato polysaccharide (PP) solutions of nominal concentrations of 200 ppm were transferred into separate test tubes. To each, 0.2 mL of freshly prepared 5% phenol solution was added. Subsequently, 1 mL of concentrated sulfuric acid was introduced rapidly and directly into each mixture. Immediate mixing was achieved using a vortex shaker to ensure homogeneity and minimize local overheating, which could lead to inconsistent colour development or sample degradation. The reaction mixtures were allowed to stand at ambient temperature (~25 °C) for a fixed period of 10 min, after which the colour had fully developed. Spectrophotometric measurements were conducted immediately following the reaction period. Each reaction mixture was then transferred into a clean plastic cuvette, and absorbance was recorded at 490 nm as per Section 2.3.3, or their photos were taken and analysed using the new smartphone-based image analysis outlined in Section 2.4.

#### 2.5.2. Preparative Size Exclusion Chromatography of Polysaccharides

Commercial pectin with the following specifications, ≥74% galacturonic acid (on a dry weight basis), <10% moisture and molecular weight of 10–300 kg/mol (2.5 g), was dissolved in 50 mL of deionized water with continuous stirring (500 rpm) at room temperature until complete dissolution. One millilitre (1 mL) of the resulting solution was loaded directly on to a Sephacryl S200HR (60 cm × 1.6 cm) size exclusion chromatographic separation system (Sigma, Gillingham, UK) and eluted with distilled water at room temperature (~25 °C) at a flowrate of 0.25 mL/min, and the total column volume of the column was estimated to be ~125 mL. A total of 26 fractions of 5 mL (F1–F26) were collected. As described in Section 2.3.4, galacturonic acid standards were prepared at concentrations of 0–200 ppm. Aliquots (20 µL) of each SEC fraction (F1–F26) and each calibration standard were transferred into individual glass vials. To each vial, 20 µL of 5% (*w*/*v*) phenol solution was added, followed by the rapid addition of 3 mL concentrated sulfuric acid.

Samples were immediately mixed using a mechanical shaker to ensure homogeneity and allowed to stand for 5 min at room temperature for colour development. One image of each sample or standard was captured under consistent lighting conditions. Quantitative colour analysis was performed using ImageJ. Images were converted to RGB format, and a central region of interest (ROI) was selected for each sample to minimize edge artifacts. Mean blue (B) intensity values were extracted (n = 3). These values were used as analytical signals for quantification. A calibration curve was constructed, using the first six vials on left of Figure 3 and plotting the measured change in blue colour against galacturonic acid concentration (0–200 ppm). Linear regression analysis was applied to obtain the calibration equation. Concentrations of galacturonic acid in SEC fractions were determined from the calibration curve. The concentration of galacturonic acid in each fraction was plotted as a function of fraction number (F1–F26) to generate the size exclusion chromatogram.

### 2.6. Statistical Analysis

In Section 2.3, Section 2.4 and Section 2.5, all the monosaccharide standards were measured in triplicate, and their means, standard deviations and calibration plots were determined using MS Excel (Microsoft Corporation, Redmond, WA, USA). Statistical information relating to the line of best fit; the slope (m), the intercept (c) and the standard deviation of the residuals (s_x/y_) were estimated using Excel’s built-in LINEST function. In Section 2.5.1, the values were measured in triplicate twice (n = 6) for each sample and the estimated values of the total carbohydrate contents were then compared (assuming equal variance), using a two-sample *t*-test (Minitab, version 19, Herndon, VA, USA), where values of <0.05 are significantly different at 95% confidence.

## 3. Results and Discussion

This study aimed to adapt the phenol–sulfuric acid (PSA) assay for use with a camera on a mobile device for the quantification of various monosaccharides. For each monosaccharide, key quantitative indicators such as the limit of detection (LOD) and limit of quantification (LOQ) were evaluated and compared with the traditional spectrophotometer-based assay. The findings confirmed that both the traditional and smartphone-based PSA methods can detect and quantify low concentrations of carbohydrates (Table 1).

### 3.1. Traditional PSA Assay

#### 3.1.1. Calibration Curves and Linearity

Calibration data were measured, and calibration curves were constructed for nine monosaccharides: glucose, fucose, xylose, fructose, galactose, galacturonic acid, rhamnose, mannose and arabinose. Each monosaccharide was measured over the concentration range of 0 to 100 ppm. Absorbance readings at 490 nm were plotted against concentration, and linear regression analysis was applied to derive the slope (m), intercept (c), and coefficient of determination (Adj. R^2^) for each standard curve.

The results revealed excellent linearity across the tested range. All calibration curves exhibited Adj. R^2^ values greater than or equal to 0.992. For example, glucose yielded an Adj. R^2^ of 0.997 with a slope of 0.0074 and an intercept of 0.0073, indicative of a highly predictable relationship between absorbance and concentration. Similar trends were observed for other sugars: fucose (Adj. R^2^ = 0.998), arabinose (Adj. R^2^ = 0.997), and fructose (Adj. R^2^ = 0.997), among others (Table 1).

The calibration curve slope reflects the assay’s sensitivity toward each monosaccharide. Steeper slopes, such as those seen for arabinose, xylose and fructose, correspond to stronger absorbance responses per unit concentration. In contrast, rhamnose, though demonstrating excellent linearity, had a slightly lower slope (0.0056), suggesting comparatively reduced colour development, possibly due to lower furfural yield under acid hydrolysis [33,34]. The sensitivity of monosaccharide detection methods, as reflected by calibration curve slopes, can vary depending on the specific assay conditions and the monosaccharide being analysed. In previous research, it has been noted that pentoses like xylose tend to produce higher yields of furfural under acid hydrolysis, compared to hexoses [15]. This is consistent with the steeper slope observed for xylose in the calibration curve. The higher furfural yield from xylose is also supported by studies on biomass conversion, where xylose-to-furfural yields are often higher than glucose-to-HMF yields [15,38].

However, the relative sensitivity of fructose compared to other sugars can vary depending on the specific assay conditions. For instance, in some studies, fructose has shown higher sensitivity in colorimetric assays due to its ketose structure, which can lead to more rapid dehydration and colour formation [39]. The lower slope for galactose, indicating reduced colour development, is consistent with its generally lower reactivity in acid-catalysed dehydration reactions compared to other monosaccharides [40].

#### 3.1.2. Sensitivity: Limits of Detection and Quantification

The limit of detection (LOD) and the limit of quantification (LOQ) are critical parameters in analytical chemistry, providing insight into the sensitivity and reliability of analytical methods. LOD and LOQ values were calculated using Equations (1) and (2).

The LOD values ranged from 5.5 ppm (galactose) to 12.3 ppm (rhamnose) (Table 1), while LOQ values ranged from 16.8 ppm to 37.5 ppm. Galactose demonstrated the highest sensitivity (lowest LOD), whereas rhamnose exhibited the highest LOD, suggesting differential assay performance depending on sugar type. Values for LOD are close to those published previously, of 0.05–15.1 ppm [2,12,17,18,19,20,21,22,23,24,25,26,41,42], as are the LOQ values.

### 3.2. Image-Based Colour Detection

#### 3.2.1. Calibration Curves and Linearity

All calibration curves exhibited Adj. R^2^ values greater than or equal to 0.991. For instance, glucose yielded an Adj. R^2^ of 0.993 with a slope of 0.9110 and an intercept of 4.1746, indicative of a highly predictable relationship between the measured change in colour intensity and concentration. Similar trends were observed for other sugars: fucose (Adj. R^2^ = 0.994), arabinose (Adj. R^2^ = 0.995), and fructose (Adj. R^2^ = 0.996), among others.

#### 3.2.2. Sensitivity: Limits of Detection and Quantification

To complement the traditional UV-Vis spectrophotometry, ImageJ software was employed to analyse the colour intensity of carbohydrate solutions captured as digital images. Red, green, and blue (RGB) values obtained from photographs were plotted against monosaccharide concentrations. It was noted that the change in blue “colour” intensity was the best for quantitative purposes (see for example Figure 2). This approach demonstrated the potential of digital image analysis as a quantitative alternative to spectrophotometry. The LOD values ranged from 7.9 ppm (rhamnose) to 13.1 ppm (mannose), while LOQ values ranged from 24.1 ppm to 39.9 ppm (Table 1). These values are not so different (~1.4× higher on average) than those obtained from the traditional method (Table 1), although in the case of rhamnose this method performed better.

The results of this study demonstrate the usefulness of the PSA assay as an analytical tool for carbohydrate quantification. Through the systematic analysis of several different monosaccharides, this study uncovered distinct differences in limits of detection and quantification which may be related to the chemical properties of these substances. As this is more a proof of concept, it is possible, if not probable, that with further optimisation the LOD/LOQ for the smartphone-based analysis will hopefully approach those obtained from spectrophotometry.

### 3.3. Factors Influencing LOD and LOQ

Multiple factors were identified that contributed to the variations in LOD and LOQ values for the different monosaccharides. First and foremost is the chemical reactivity of each monosaccharide. The formation of furfural or hydroxy-methyl furfural, the key intermediates that react with phenol to form the chromophore, depends on the sugar’s ability to undergo dehydration [43]. In addition, absorbance properties at 490 nm are not uniform across all sugars [13]. Although the PSA assay is typically conducted at this wavelength, some sugars may have absorption maxima slightly shifted due to their specific chromophore [26]. Suboptimal absorbance at 490 nm could result in lower sensitivity for certain sugars [3] and the same would be true for any colour measured using ImageJ.

### 3.4. Practical Applications and Significance

The PSA assay demonstrated in this study is well-suited for applications requiring the detection and quantification of low-concentration carbohydrates. These include, but are not limited to, trace sugar analysis in food and beverage quality control, assessment of monosaccharide composition in biomass hydrolysates, and monitoring of metabolic intermediates in microbial fermentation [3,44]. The assay’s simplicity, affordability and sensitivity may be valuable in contexts where advanced instrumentation is either unavailable or impractical [45]. The success of ImageJ-based colour detection underscores the growing potential of integrating digital tools with traditional analytical chemistry [30,31,32]. The ability to quantify colour intensity (or changes in colour intensity) from smartphone images and correlate it with concentration has the potential to introduce new possibilities for decentralized and/or rapid testing, in, for example, field research or educational settings [45,46,47,48,49,50,51,52,53,54,55]. It is also possible that in the future that this approach could be extended/adapted to other areas of biopolymer characterisation which rely on colorimetric analysis, for example, to quantify protein content (Bradford assay), uronic acid content (carbazole assay) or total polyphenol content (Folin–Ciocalteu assay).

To demonstrate the potential of this new method, two case studies were developed, one to estimate the total carbohydrate content of extracted polysaccharides compared to the traditional PSA, and one to generate the size exclusion chromatogram of a commercial citrus pectin sample.

#### 3.4.1. Total Carbohydrate Content of Polysaccharides

The phenol–sulfuric acid assay is used extensively in carbohydrate chemistry to determine the total carbohydrate contents of polysaccharides [3,13,16] and in most cases, unless there is any further information available, glucose is used a standard [36,37]. In this short case study, the total carbohydrate content of pectin-like polymers from okra and potato (Table 2) were estimated using both the traditional and the new smartphone-assisted phenol–sulfuric acid assay with ImageJ analysis methodology described in Section 2.5.1.

The values using the new smartphone-based assay are on average a little higher for all samples; the values are close (within 15%), although due to the large number of replicates (n = 6) these differences are often significant (*p* < 0.05) from a statistical point of view. Considering the widespread availability of smartphones, it seems reasonable to propose that this new smartphone-assisted phenol–sulfuric acid assay with ImageJ analysis could have potential in this area. One possible explanation for the estimated differences in the total carbohydrate contents between samples is that different monosaccharides react differently to the assay (Table 1) and produce “colour” of different intensities at their wavelength of maximum absorbance (*λ_max_*), which may vary slightly from 490 nm [3,13].

#### 3.4.2. Preparative Size Exclusion Chromatography of Citrus Pectin

One advantage of this new smartphone-based assay is the potential to analyse multiple samples in one image. To demonstrate this, a commercial citrus pectin sample was separated on a preparative size exclusion column, and an aliquot of each fraction was analysed using this new approach. Figure 3 shows the raw data, i.e., the image captured by the smartphone camera and the size exclusion chromatogram after data analysis. As can be seen from Figure 3 this allowed the analysis of 32 samples (in triplicate) in one image. This is particularly useful where many samples are produced or to researchers in resource-limited environments.

## 4. Conclusions

This study evaluated a novel smartphone-assisted phenol–sulfuric acid assay with ImageJ analysis for total carbohydrate quantification across a broad concentration range. The key findings can be summarized as follows:

- The integration of ImageJ software for digital colour analysis offers a quantitative alternative to spectrophotometry. LODs and LOQs using this novel method were 7.9–13.1 and 24.1–39.9 ppm, respectively, which were not very different from the traditional method (5.5–12.3 ppm (LOD) and 16.8 and 37.5 ppm (LOQ)).
- The assay exhibited excellent linearity (Adj. R^2^ ≥ 0.991) for all tested monosaccharides.
- Differences in LOD and LOQ between monosaccharides were related to variations in chemical characteristics.

These results represent a potential alternative to the traditional PSA protocols and could have potential in areas such as food quality control, bio-industrial research and polysaccharide quantification. Future research should also explore automation and greater integration with chromatographic techniques.

## Figures and Tables

**Table 1:** Quantitative analysis of monosaccharide content in sample solutions, using the phenol–sulfuric acid assay method. Each monosaccharide was measured at six concentrations (0, 20, 40, 60, 80, and 100 ppm), and in three independent measurements.

Monosaccharide	Traditional Method					Novel Smartphone-Based Method				
	Adj. R^2^	Slope	Intercept	LOD (ppm)	LOQ (ppm)	Adj. R^2^	Slope	Intercept	LOD (ppm)	LOQ (ppm)
Glucose	0.997	0.0074	0.0073	7.0	21.3	0.993	0.9110	4.1746	11.8	36.0
Fucose	0.998	0.0086	0.0200	6.7	20.4	0.994	0.9805	2.1905	10.5	32.0
Xylose (see Figure 2)	0.998	0.0091	0.0233	6.6	20.1	0.994	0.9881	1.6508	11.1	33.8
Fructose	0.997	0.0100	0.0225	7.5	22.9	0.996	0.9900	0.3140	8.5	25.9
Galactose	0.998	0.0072	0.0014	5.5	16.8	0.994	0.8824	4.0476	11.1	33.8
Galacturonic Acid	0.995	0.0071	0.0102	6.5	19.8	0.994	0.9710	1.2698	10.3	31.4
Rhamnose	0.992	0.0056	0.0031	12.3	37.5	0.997	0.7095	1.5238	7.9	24.1
Mannose	0.996	0.0075	0.0027	8.1	24.7	0.991	1.1419	0.3175	13.1	39.9
Arabinose	0.997	0.0096	0.0260	7.3	22.3	0.995	1.4633	4.7778	10.2	31.1

**Table 2:** Quantitative analysis of the total content in okra polysaccharide (OP) and potato (PP) solutions using the phenol–sulfuric acid (PSA) assay.

Sample	Total Carbohydrate Content (ppm) − Traditional PSA Assay	Total Carbohydrate Content (ppm) − Novel Smartphone-Based PSA Assay	*p*-Value from Two-Sample *t*-Test
OP1	124 ± 4	143 ± 6	0.000
OP2	135 ± 5	142 ± 11	0.169
OP3	133 ± 6	150 ± 7	0.001
OP4	137 ± 6	149 ± 7	0.009
OP5	138 ± 1	131 ± 6	0.020
OP6	147 ± 14	135 ± 5	0.076
OP7	138 ± 3	144 ± 5	0.033
OP8	147 ± 7	132 ± 1	0.002
OP9	137 ± 3	149 ± 9	0.014
OP10	134 ± 9	150 ± 13	0.024
OP11	136 ± 11	152 ± 19	0.105
Overall average OP (n = 66)	137 ± 9	143 ± 11	0.000
PP1	142 ± 3	163 ± 2	0.000

The values represent the mean ± one standard deviation (n = 6). Results were then compared using a two-sample *t*-test, where values of <0.05 are significantly different at 95% confidence.

## Data Availability

The original contributions presented in this study are included in the article. Further inquiries can be directed to the corresponding author.
